# Automatic Detection of Atrial Fibrillation Based on Continuous Wavelet Transform and 2D Convolutional Neural Networks

**DOI:** 10.3389/fphys.2018.01206

**Published:** 2018-08-30

**Authors:** Runnan He, Kuanquan Wang, Na Zhao, Yang Liu, Yongfeng Yuan, Qince Li, Henggui Zhang

**Affiliations:** ^1^School of Computer Science and Technology, Harbin Institute of Technology, Harbin, China; ^2^School of Physics and Astronomy, The University of Manchester, Manchester, United Kingdom; ^3^Space Institute of Southern China, Shenzhen, China; ^4^Key Laboratory of Medical Electrophysiology, Ministry of Education, Collaborative Innovation Center for Prevention and Treatment of Cardiovascular Disease, Institute of Cardiovascular Research, Southwest Medical University, Luzhou, China

**Keywords:** atrial fibrillation, continuous wavelet transform, 2D convolutional neural networks, time-frequency features, practical applications

## Abstract

Atrial fibrillation (AF) is the most common cardiac arrhythmias causing morbidity and mortality. AF may appear as episodes of very short (i.e., proximal AF) or sustained duration (i.e., persistent AF), either form of which causes irregular ventricular excitations that affect the global function of the heart. It is an unmet challenge for early and automatic detection of AF, limiting efficient treatment strategies for AF. In this study, we developed a new method based on continuous wavelet transform and 2D convolutional neural networks (CNNs) to detect AF episodes. The proposed method analyzed the time-frequency features of the electrocardiogram (ECG), thus being different to conventional AF detecting methods that implement isolating atrial or ventricular activities. Then a 2D CNN was trained to improve AF detection performance. The MIT-BIH Atrial Fibrillation Database was used for evaluating the algorithm. The efficacy of the proposed method was compared with those of some existing methods, most of which implemented the same dataset. The newly developed algorithm using CNNs achieved 99.41, 98.91, 99.39, and 99.23% for the sensitivity, specificity, positive predictive value, and overall accuracy (ACC) respectively. As the proposed algorithm targets the time-frequency feature of ECG signals rather than isolated atrial or ventricular activity, it has the ability to detect AF episodes for using just five beats, suggesting practical applications in the future.

## Introduction

Atrial fibrillation (AF) is recognized as a major cardiovascular disease, affecting a large number of the population (Zoniberisso et al., 2014; Potter and Le, 2015). AF is associated with increased risks of cardiovascular events, reducing the life quality of AF patients or even causing mortality (Hylek et al., 2003; Mathew et al., 2009). AF is also related to obesity, long-term alcoholism and obstructive sleep apnea, each of which promotes the development of AF (Gami et al., 2004; Mukamal et al., 2005; Miyasaka et al., 2006; Mathew et al., 2009, p. 25). Furthermore, the lack of a deep understanding for its pathophysiological mechanisms affects the diagnosis of AF (Censi et al., 2013). Therefore, an early detection of AF appears to be important for effective treatments of AF.

Based on the duration of episodes, AF can be classified into three main types, namely paroxysmal, persistent, and permanent (January et al., 2014). Paroxysmal AF is usually the primary condition of the arrhythmia, with which the episodes terminate spontaneously within 7 days after its initiation; whilst persistent and permanent AF can last for more than several months. For many AF patients they may initially suffer very short episodes, but the episodes increase in frequency and duration, leading to be persistent by a mechanism of AF begetting AF (de Vos et al., 2010). For this condition, AF may last longer than 7 days, to terminate which one may need interventions such as pharmacological or electrical cardioversion. Without treatment, persistent AF may turn into permanent AF, one of the most sustained cardiac arrhythmias (Zoniberisso et al., 2014).

Early detection of AF is essential for effective treatments. However, it is still not easy to address the early AF detection task though the use of long-term ECG recording devices is available. Proximal AF episodes often last only for a few beats in duration, therefore, it is very time-consuming to detect AF by visual inspection (Dash et al., 2009). Such a challenge calls for a wide variety of automatic AF detectors. For the past years, a series of sophisticated methods have been developed to tackle the challenges of AF detection (Kikillus et al., 2007; Couceiro et al., 2008; Babaeizadeh et al., 2009; Yaghouby et al., 2010; Larburu et al., 2011; Parvaresh and Ayatollahi, 2011). Two classes of AF detection methods, the atrial activity analysis-based (Artis et al., 1991; Slocum et al., 1992; Lake and Moorman, 2011; Zhou et al., 2014; Ladavich and Ghoraani, 2015) and the ventricular response analysis-based (Moody and Mark, 1983; Tateno and Glass, 2001; Dash et al., 2009; Park et al., 2009; Huang et al., 2011; Lian et al., 2011; Yaghouby et al., 2012; Lee et al., 2014) method, attract the interest of the most of studies. The first category methods utilize the absence of P waves or the presence of f-waves for diagnosis. The performance of this kind of method highly depended on the signal quality, which is hard to be guaranteed in the practice. The second category methods are based on the variability of RR intervals. Although these kinds of methods have a robust noise resistance, its diagnosis accuracy is unsatisfactory when a wide variety of rhythms need to be dealt with due to the limitation of the information conveyed by RR intervals (Petrutiu et al., 2006; Huang et al., 2011; Lian et al., 2011; Lee et al., 2014).

Over past years, algorithms based on convolutional neural networks (CNNs) have proved successful in information classification in many fields, such as object detection, speech, and image recognition (Lecun et al., 2015). However, CNNs-based algorithms for stratifying cardiovascular diseases are not well-established due to limited availability of ECG database. Though a few previous studies have applied CNNs to detect cardiac arrhythmias (Rajpurkar et al., 2017; Vollmer et al., 2017), it still remains a challenge to develop an effective algorithm for detecting AF based on short episodes of ECGs.

The objective of this study was to address some potential drawbacks of existing AF classification methods by developing an accurate and reliable one for the fully automated classification of AF based on continuous wavelet transform (CWT) (Addison, 2005) and 2D CNNs (Krizhevsky et al., 2012) methods. Such possible drawbacks of traditional AF classification methods include: (i) manual extraction of ECG features that limits the accuracy of classification; (ii) low efficacy for fast AF detection with a short period of ECG signal data; and (iii) the use of atrial or ventricular only activities to classify AF and normal condition, lacking consideration of complete information of ECG signal. The advantage of the proposed algorithm over others lies at that we do not need to manually extract features of ECG signals. Instead, the proposed CNNs can automatically extract the spatio-temporal features of ECG patterns obtained by the CWT analysis with proper trainings. In addition, the proposed algorithm can detect AF by using only five beats to achieve a significant performance, suggesting potentials for clinical applications.

The developed method was tested and validated by the MIT-BIH Atrial Fibrillation Database (AFDB) (Goldberger et al., 2000).

The rest of this paper is organized as follows. In Section “Materials and Methods,” the method of three-stage AF classification is described in details. In Section “Implementation of the Algorithm,” some details about the implementation of the algorithm are presented. In Section “Results and Discussion,” the proposed method is evaluated using the AFDB, and its performance with varied CNNs parameters is analyzed. AF detection results by the presented method are compared with those from other existing algorithms. Finally, Section “Conclusion” concludes our study.

## Materials and Methods

**FIGURE 1:**
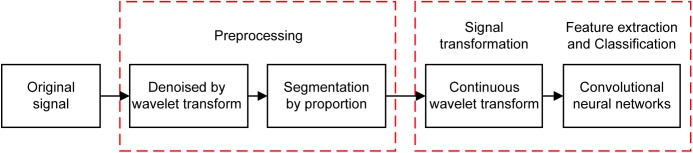
Flowchart diagram of the proposed AF detection algorithm. Original ECG data is first preprocessed for denoising and segmentation, then CWT is applied to transform time series of ECG signals into 3D patterns which is further classified by trained CNNs.

The flowchart of the proposed AF detection method is shown in **Figure 1**. It includes four stages in two phases: phase 1 is for pre-processing (data denoising and data segmentation) and phase 2 for CWT and AF classification with CNNs. In phase 1, the wavelet transform (WT) method is applied to remove the noise from the ECG signal, which is then segmented into a series of periods, each of which has duration of 1.2 s (i.e., 300 sample points given the sampling rate of 250 Hz). In phase 2, CWT is first employed to transform the five beats of the ECG signal in each segment to a 3D time-frequency representation of ECG patterns. Then, the proposed CNNs was properly trained to process the AF classification.

### Database

The MIT-BIH AFDB was used to evaluate the performance of the developed AF detection method. The database contains 25 ECG recordings with about 10-h in duration length mainly from PAF patients, which were obtained with a 250 Hz sampling rate. However, in the present study four recordings in database were excluded because two raw recordings (“00735” and “03665”) are not available, and the other two recordings (“04936” and “05091”) have some incorrect reference annotations. The database contains 605 episodes for four different rhythms, among them 291 episodes are for AF, 14 episodes for atrial flutter, 12 episodes for junctional rhythm, and 288 episodes for other rhythms. For each ECG recording, it contains ECG signals from two leads, first of which was used for this study.

### Noise Filtering

Raw data of ECG is contaminated by noises. Therefore, the WT method (Shyu et al., 2004; Gomes et al., 2010) is used to filter noises.

For a time series of ECG f(t), its WT with respect to a given mother wavelet (ψ) is defined as the following (Shyu et al., 2004; Gomes et al., 2010):

(1) $$W_{\text{a},\text{b}}f(t)=\frac{1}{|a{|}^{1/2}}\int_{\infty }^{\infty }f(t)\psi \left(\frac{t-b}{a}\right)\mathrm{dt}$$

where *a*, *b* and *W*_a,b_ are the scale factor, translational value and WT respectively. Letting *a* = 2^j^ (j∈Z,Z is the integral set), the WT is regarded as dyadic WT, which represents better the multiscale characterization of ECG signals than the CWT does (Mallat and Zhong, 1992). For a discrete time series of *f(t)*, which is denoted as *f(n), n = 1, 2, 3 …N*, the calculation of dyadic WT is derived from Equation (1) for low and high frequency components as represented as:

(2) $$S_{2^{\text{j}}}f(n)=\sum_{\text{k}\in \text{Z}}h_{\text{k}}S_{2^{\text{j}-1}}f(n-2^{\text{j}-1}k)$$

(3) $$W_{2^{\text{j}}}f(n)=\sum_{\text{k}\in \text{Z}}g_{\text{k}}S_{2^{\text{j}-1}}f(n-2^{\text{j}-1}k)$$

where *S*_2^j^_ and *W*_2^j^_ are the smoothing operator of the WT, and *h*_k_ and *g*_k_ are the coefficients of the corresponding high and low filters. In this study, we decomposed original ECG signal into seven scales (the corresponding frequency bands are 62.5–125, 31.25–62.5, 15.63–31.25, 7.81–15.63, 3.91–7.81, 1.95–3.91, and 0.98–1.95 Hz, respectively). By using the *Daubechies4 (db4)* wavelet function (Daubechies, 1988), the input ECG signal *f(n)* is decomposed into low frequency and high frequency components, and the low frequency component is put into the next layer for further decomposition. The reason why we choose the *db4* wavelet is due to its good regularity, which makes the reconstruction of ECG signals smooth (Daubechies, 1988).

**FIGURE 2:**
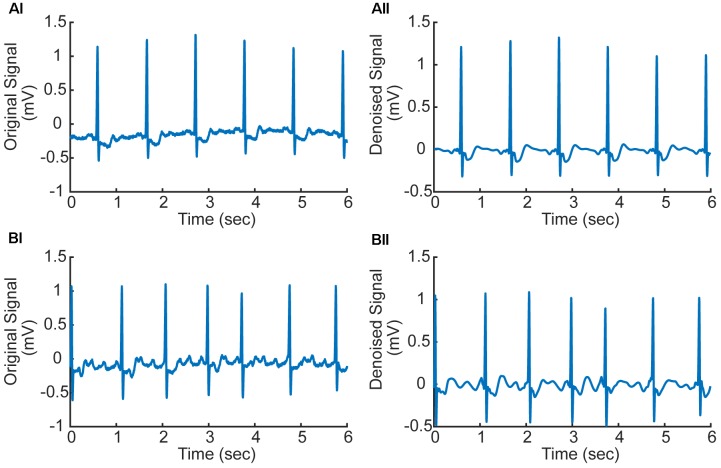
**(Ai,Aii)** Original and denoised ECG signal in AF condition (ECG record 07910). **(Bi,Bii)** Original and denoised ECG signal in normal condition (ECG record 07910). f-Waves between two consecutive R-waves are apparent illustrating atrial fibrillation (AF).

In numerical implementation, it was found that the high frequency noise was mainly determined by one to three scale bands. Therefore, the values of these three scales were set to zeros to filter the high-frequency noise. The filtered signals were used in subsequent processing, which are shown in **Figure 2** for AF and normal ECG signals.

### Data Segmentation by Proportion

Segmentation of ECG signal into a series of time periods usually requires precise detection of boundaries and peak positions of the three characteristic of ECG waves (i.e., P, QRS, and T waves corresponding to the depolarization of the atria and ventricles, and repolarization of the ventricles respectively), which are termed as fiducial points. In this study, segmentation of ECG recordings into a series of time windows was based on the annotated R-peak locations of the database, allowing us to directly compare our results with the performance of other existing methods.

**FIGURE 3:**
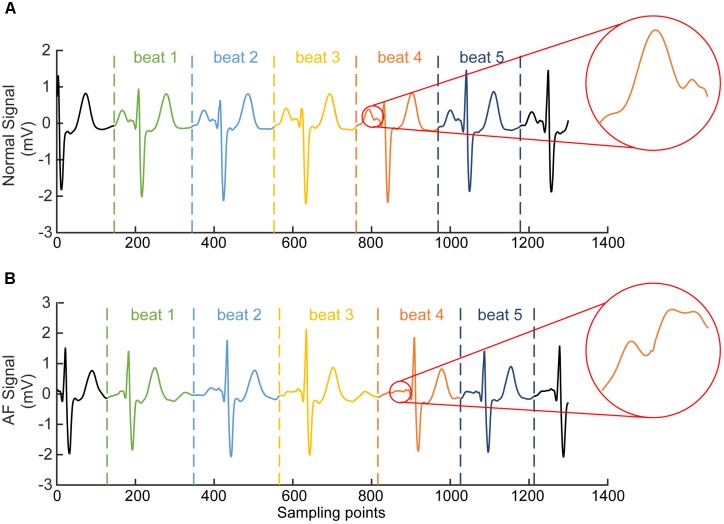
Illustration of segmented ECG signals into five beats for normal **(A)** and AF **(B)** conditions. Each beat was marked by two dotted lines with the same color. In **(A)** for normal ECG, a clear normal P wave is present as shown by the encircled inset. In **(B)** for AF ECG, abnormal f-waves are apparent as shown in the encircled inset.

For each segmented time window of the ECG signal, it contains one heart beat cycle and has a length of 1.2 s (i.e., about 300 sampling points), starting at the 2/3 period of the previous RR interval (which is the interval between the peaks of the previous QRS complex to the current QRS peak, see **Figure 3**). Each segment contains the information of atrial and ventricular activities. The reason for the segment size to be chosen is to allow each segment contain most of the information in one heart beat cycle for both AF and normal conditions. An example of segmented ECG signals into five beats for AF and normal conditions are shown in **Figure 3**, with each beat being marked by two dotted lines with the same color. For normal ECG signal (**Figure 3A**), a clear P wave is present as shown by the encircled inset. For AF ECG signal (**Figure 3B**), no clear P wave is present. Instead, a series of continuous, rapid and irregular f-waves is present indicating AF (f-waves) (see the encircled inset).

### Continuous Wavelet Transform

Feature extraction plays a key role for AF classification (Chazal and Reilly, 2006). Any information in the ECG signals that can be used to discriminate AF from normal condition is considered as one feature. The features can be extracted in various forms directly from the processed ECG signal in the time, frequency and time-frequency domains.

Previous studies have investigated various ways to extract ECG features, among them the WT methods are believed to be the most efficient for processing ECG signals (Guler and Ubeyli, 2005; Lin et al., 2008; Kutlu and Kuntalp, 2012). By WT, one can extract ECG information in both frequency and time domains, which is superior to the traditional Fourier transform, which can only analyze ECG information in the frequency domain (Dokur and Olmez, 2001). For various types of WT (Addison, 2005), the most popular one for ECG classification is the discrete wavelet transform (DWT) (Kozak et al., 2008). In addition to DWT, CWT has also been used to extract features from the ECG signals, since it solves many of DWT defects, such as the coarseness of the representation and instability, which has been applied successfully for at least a decade ago (Addison, 2005).

**FIGURE 4:**
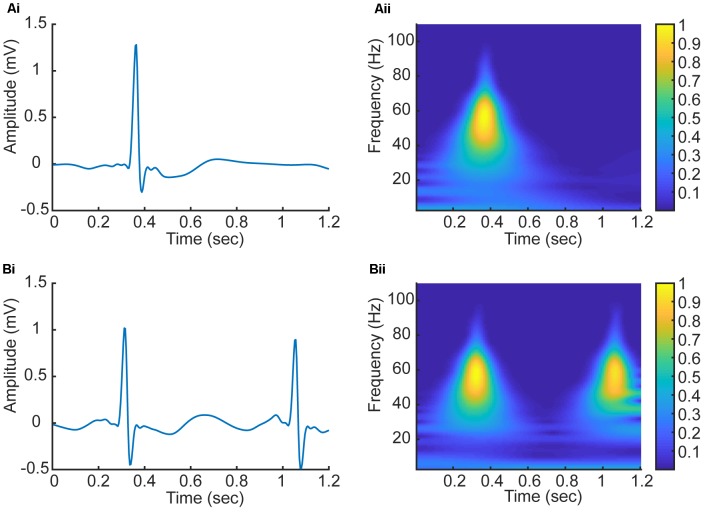
**(Ai,Aii)** 1D time domain signal and 2D frequency-time pattern based on CWT in AF condition. **(Bi,Bii)** 1D time domain signal and 2D frequency-time pattern based on CWT in normal condition.

In this study, we have employed CWT based Equation (1) with the *Daubechies5 (db5)* wavelet to transform five beats to a series of five corresponding 2D CWT patterns, which can be regarded as a 3D time-frequency representation of ECG signals (Daubechies, 1988). **Figure 4** shows examples of the 2D patterns of CWT transformation from normal and AF ECG signals. In the figure, the color codes the density of the signal component in the corresponding frequency with brighter color representing a higher density. As shown in **Figures 4Ai,Aii**, there are some differences in the 1D time series of ECGs between normal and AF conditions. First, there is no clear or a dominant P wave in the AF ECG (**Figure 4Ai**) as compared to the normal ECG (instead, high frequency but low amplitude f-waves exist). Second, due to the filtering effect of atrioventricular node, there is no 1:1 ventricular response to the atrial excitation, therefore the RR interval is different between the AF and normal conditions. Consequentially during each segmented time window of the ECG signal (fixed for 1.2 s, i.e., 300 sampling points), one R wave presents in the AF ECG (**Figure 4Ai**) whilst two R waves presents in the normal ECG (**Figure 4Aii**). Such different information in the time domain are reflected by the CWT, which converts the 1D time domain signal into the 2D pattern in the time-frequency domain of the distribution of frequency at different timings, which can be used to better differentiate AF from normal condition. The 2D CWT patterns for AF and normal conditions are shown in **Figures 4Aii,Bii** correspondingly.

### The Basic Construction of Convolutional Neural Networks

Convolutional neural network has been shown to be able to automatically extract features of signals without any data pre-processing and pre-training algorithms (Arel et al., 2010). A traditional CNNs is composed of an input and an output layer, as well as multiple hidden layers which typically consist of convolutional layers, pooling layers, and fully connected layers (Hubel and Wiesel, 1959).

For convolutional layers, they are locally connected to extract and convolve the features by applying a set of weights which are called filter or kernel (Hubel and Wiesel, 1959). Basically, the relevant high-level features can be extracted with the increase of the number of the convolutional layers. The weights of the parameters of the convolutional kernels in each layer are trained with the backpropagation (BP) error algorithm (Rumelhart et al., 1988). For an activation function, a sigmoid function is usually applied to the convolved features as follows:

(4) $$\alpha_{\text{i}}^{k}=W^{k}x_{\text{i}}+b^{k}$$

(5) $$\beta_{\text{i}}^{k}=\frac{1}{1+e^{-\alpha_{\text{i}}^{k}}}$$

where $\alpha_{\text{i}}^{k}$ represents the convolution result that is for the ith input and the kth feature map, *W^k^* and *b^k^* respectively represent the corresponding weights and bias terms for the *kth* feature map. The sigmoid function output $\beta_{\text{i}}^{k}$ is applied to the *kth* feature map producing outputs. Furthermore, *x_i_* denotes the *ith* training data which is an n-dimension vector.

**FIGURE 5:**
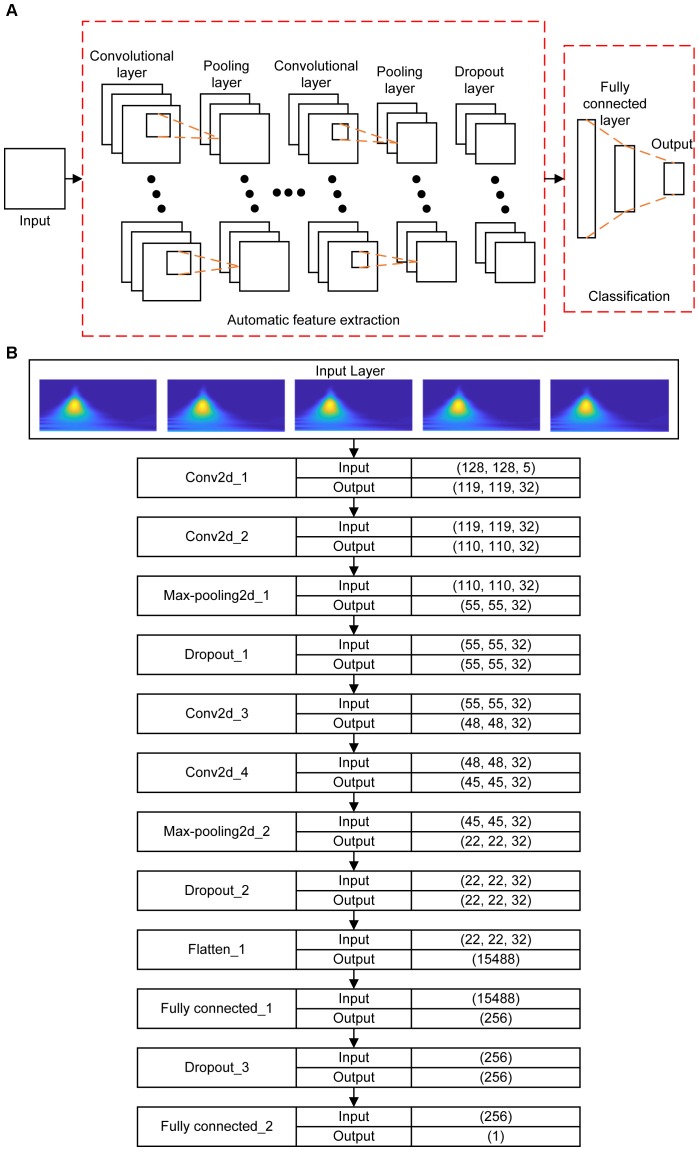
**(A)** Schematic illustration of the basic construction of CNNs. **(B)** The architecture of the proposed CNNs. The input instance of the proposed CNNs is five 128 × 128 images representing the CWT spectrums of corresponding five beats. In the convolutional layer, the output size is denoted as (x, y, z), where (x, y) is the size of the feature map in this layer, and z is the number of convolutional kernels. In the Max-pooling layer, the size of the feature map is reduced by half in both x and y axes. In the Dropout layer, the output size is the same as the output of previous layer. In the Flatten layer, the output is flattened to an 1D vector. In the Fully connected layer, the output size is the same as the number of neurons of the layer.

After the convolution layer, the dimensionality of the extracted features is reduced in order to improve the speed of the training process, so the pooling layer is applied to the following hidden layer which is called the subsampling layer merging similar features into one. The action of the pooling layer is to compute the averaged convolved features within the neighboring neurons that are laid in the prior convolutional layer. In some cases, the dropout layer may be applied for random training network parameters to prevent over-fitting. After a given set of convolutional, pooling and dropout layers, one or more fully connected layers are employed whose neurons are jointed to the whole neurons from the previous layer at the end of the constructed CNNs. The major of CNNs parameters are generally produced by the fully connected layer parameters, which complete the mission of AF classification and determine the final classification results. The basic construction of CNNs is shown in **Figure 5A** including the common input convolutional, pooling, dropout, fully connected, and output layers.

In this paper, the CNNs architecture including the input, convolutional, max-pooling, dropout, flatten, fully connected, and output layers are designed in which the optimal parameters are described in Section “Implementation of the Algorithm.” The whole construction of the proposed CNNs for AF classification is also demonstrated in Section “Implementation of the Algorithm.”

## Implementation of the Algorithm

### The Division of Dataset

The original annotated time series of ECG signal is divided into a series of five segments, each of which contains one heart beat cycle. In total, 162,536 five beat data segments were used, among which 61,924 and 100,612 segments are for AF and normal condition respectively. It is obvious that the AF and normal samples are not balanced. To address this issue, we randomly selected the same number data of AF and normal conditions, which were assigned into training and test sets from different recordings, by which over-fitting resulted from training and testing data set from the same patient was also avoided. Eventually, we extracted a total of 100,000 samples into a training set and test set based on the proportion of 4:1, among which 80000 training and 20000 test data were used to build the model.

### The Architecture of the Proposed CNNs

Continuous wavelet transform was used to transform the five beat time domain signals into time-frequency domain signals with the *db5* wavelet (Daubechies, 1988), resulting in five corresponding 2D patterns of frequency density as a 3D input instance to the proposed CNNs.

With a 3D input instance, the structure of the proposed CNNs is designed as shown in **Figure 5B**. The CNNs structure contains four convolutional layers, three dropout layers, two max-pooling, two fully connected layers, and one flatten layer. Each of the first two convolutional layers has 32 convolution kernels with the kernel size of 10 × 10. After the convolution operations, the first max-pooling layer with pooling size of 2 × 2 was used to reduce the size of the previous output followed by a dropout layer to suppress the complexity of the network. The number of kernel of the following two convolutional layers is the same as in the previous ones, but the sizes of the kernels are 8 × 8 and 4 × 4 respectively. Followings are the second max-pooling layer with the same pooling size as the first one and a dropout layer. After these operations, the output data is transformed to a 1D vector by a flatten layer, and then feed into two fully connected layers that have 256 and 1 neurons respectively, between which is a dropout layer.

In the CNNs, the learning rate, momentum and weight decay rates are set to 0.001, 0.8, and 10^−6^ respectively. To optimize these parameters, the stochastic gradient decent (SGD) algorithm (Rumelhart et al., 1988) was implemented. Furthermore, instead of large-size convolutional kernel, a multi-layer with small-scale convolutional kernel is used in order to reduce the number of parameters and increase the non-linearity of the network.

As shown in **Figure 5B**, the Conv2d_i (*i* = 1, 2, 3, 4) represent the four convolutional layers, which are followed by two Max-pooling2d_j (*j* = 1, 2) and Dropout_k (*k* = 1, 2) layers. In this paper, the input instance of the proposed network is a 3D vector with a size of (128, 128, 5) specifying the length, width and beat number of the CWT pattern. After four convolution and two max-pooling operations, the size of the instance is transformed into a pattern of (22, 22, 32), which specify the size of convolutional result of each kernel and the number of kernels in the last convolutional layer. Through the flatten layer, the output size of the previous layer is changed to 15488 (22 × 22 × 32), which is then input into the first Fully connected_1 layer containing 256 neurons. Finally, with the third Dropout_3 and the second Fully connected_2 layer, the classification results are decided.

## Results and Discussion

The proposed method was applied to the dataset of AFDB. From classification results, we calculated four parameters: correct AF classification number [true positives *(TPs)*], false normal classification number [false negatives *(FNs)*], correct normal classification number [true negatives *(TN)*], and false AF classification number [false positives *(FPs)*]. In order to evaluate the performance of the proposed classification algorithm, the sensitivity *(Se)*, specificity *(Sp)*, positive predictive value *(PPV)*, and overall accuracy *(ACC)* were calculated using the following equations respectively.

(6) $$\mathrm{Se}=\frac{\mathrm{TP}}{\mathrm{TP}+\mathrm{FN}}\times 100\%$$

(7) $$\mathrm{Sp}=\frac{\mathrm{TN}}{\mathrm{TN}+\mathrm{FP}}\times 100\%$$

(8) $$\mathrm{PPV}=\frac{\mathrm{TP}}{\mathrm{TP}+\mathrm{FP}}\times 100\%$$

(9) $$\mathrm{Acc}=\frac{\mathrm{TP}+\mathrm{TN}}{\mathrm{TP}+\mathrm{FP}+\mathrm{TN}+\mathrm{FN}}\times 100\%$$

In this study, the newly developed algorithm using CNNs with the AFDB have achieved 99.41, 98.91, 99.39, and 99.23% for the sensitivity, specificity, PPV, and overall accuracy respectively, which was better than most of other existing algorithms as detailed below.

### Selection of Parameters

To obtain an optimal CNNs network structure to classify AF, the impacts of varied structural and training parameters of the network on output results were evaluated. By comparing the final error values of testing samples, a set of final CNNs parameters were obtained to achieve the minimum testing error, which are shown in **Table 1**.

**Table 1:** Optimal CNNs parameter set for AF arrhythmias classification.

The CNNs parameter	Value
Learning rate initial value	0.001
Moment coefficient	0.8
First convolutional layer kernel size	10
Weight decay rates	10^−6^
Second convolutional layer kernel size	10
First max-pooling layer kernel size	2
Third convolutional layer kernel size	8
Forth convolutional layer kernel size	4
Max-pooling layer kernel size	2
The number of neurons in the first fully connected layer	256
The number of neurons in the second fully connected layer	1
Epoch number	50

The number of feature maps in the pooling layers is the same as the convolutional layers. The learning rate and moment are initialized at 0.001 and 0.8 during the training procession. The classification loss function about the training samples is stable after 50 epochs of CNNs. Because of the number of the training samples, the accuracy rate of the proposed method cannot be further increased by changing the number of convolutional and max-pooling layers.

In the following analysis, the impacts of different training and structural parameters about the proposed classification method are discussed.

#### Learning Rate

In this case, the original value of the learning rate is changed systematically from 0.0007 to 0.005. **Table 2** shows the testing samples classification accuracy of different learning rate values after 50 epochs. As the performance displayed in **Table 2**, results of the proposed CNNs are not improved whether the learning rate is increased or decreased. Therefore, the optimum value of the learning rate is set to 0.001.

**Table 2:** Various learning rate for proposed CNNs.

Learning rate value	Testing samples classification accuracy (%)
0.0007	99.08
0.0008	99.12
0.0009	99.06
0.001	99.23
0.003	99.08
0.005	99.04

#### Momentum Coefficient

In this case, the influences of altering the momentum coefficient on the results of the proposed CNNs are investigated. The classification accuracy about the testing samples during 50 epochs are calculated and presented in **Table 3** with the same CNNs structural parameters. As the performance shown in **Table 3**, the results of the proposed CNNs are not improved whether the momentum coefficient is increased or decreased. Therefore, the optimum momentum coefficient value is set to 0.8. About the momentum coefficient, it can avoid the neural network into a local minimum. However, it also may result in the unstable of the network structure when the value is set quite high.

**Table 3:** Various moment coefficient for proposed CNNs.

Moment coefficient	Testing samples classification accuracy (%)
0.7	99.18
0.75	99.15
0.8	99.23
0.85	99.06
0.9	99.16
0.95	98.90

#### CNNs Structural Parameters

In this study, the final values of the learning rate and momentum coefficient are respectively set to 0.001 and 0.8. Furthermore, the changes for the other CNNs structural parameters (the convolutional and max-pooling layer kernel size and the number of neurons in the fully connected layer max-pooling layer kernel size) also have been carried out based on experimentation experiences. According to the results, the performance is not improved for different structural parameter sets, as such the parameters shown in **Table 1** are still as optimum choice for proposed CNNs.

### Comparison With Other Related Studies for AF Classification

In order to evaluate the performance of the proposed classification method, the proposed algorithm was compared with other existing algorithm employing five measurements containing the sensitivity, specificity, PPV, overall accuracy and the length of the window (WL) respectively. Many of the algorithms (Moody and Mark, 1983; Cerutti et al., 1997; Tateno and Glass, 2001; Logan and Healey, 2005; Couceiro et al., 2008; Babaeizadeh et al., 2009; Dash et al., 2009; Huang et al., 2011; Lake and Moorman, 2011; Asgari et al., 2015; Ladavich and Ghoraani, 2015; García et al., 2016; Xia et al., 2018) were chosen for comparison as the best performing results for various methods.

As shown in **Table 4**, the performance of the proposed method is better than all of other algorithms in comparison for classifying AF which implemented on the same database of AFDB. In addition, the present algorithm is capable of AF detection by using only five beats, which is superior to other algorithms. This is due to the implementation of finer CWT transform and better designed CNNs network of the present algorithm as compared to the others, allowing a more accurate identification of AF by using shorter ECG segments based on automatically extracting pattern features of 2D CWT transformation patterns.

**Table 4:** Comparison of the performances of AF classification algorithms based on the same database of AFDB.

Algorithm	Year	WL (s)	Se (%)	Sp (%)	PPV (%)	ACC (%)
Moody and Mark	1983	60	87.54	95.14	92.29	92.12
Cerutti et al.	1997	90	96.10	81.55	75.76	83.38
Tateno and Glass	2001	50	94.40	97.20	96.10	–
Logan and Healey	2005	120	96.00	89.00	–	–
Couceiro et al.	2008	60	93.80	96.09	–	–
Babaeizadeh et al.	2009	>60	89.00	96.00	88.00	–
Dash et al.	2009	128 beats	94.40	95.10	–	–
Lake and Moorman	2011	12	91.00	94.00	–	–
Huang et al.	2011	101 beats	96.10	98.10	–	–
Ladavich and Ghoraani	2015	7 beats	98.09	91.66	79.17	93.12
Asgari et al.	2015	9.8	97.00	97.10	–	97.10
Garcia et al.	2016	7 beats	91.21	94.53	–	93.32
Xia et al.	2018	5	98.79	97.87	–	98.63
Proposed Algorithm	2018	5 beats	99.41	98.91	99.39	99.23

### Clinical Relevance

The proposed method is able to classify AF with five beat cycles. It has potentials to be used as an assistant diagnosing tool for clinic uses. In general, AF is the main causes of strokes so it is essential to diagnose it in the early stage for AF patients. Upon a proper early diagnosis, efficient treatment plans like rate-control medication or anticoagulation therapy for AF patients can be made to reduce the occurrence of strokes. As the proposed algorithm requires only five beats, proximal AF data can be detected as AF with a high accuracy.

## Conclusion

We developed a framework based on time-frequency representation of ECG signals and CNNs architectural model for automated classification of AF. The framework transforms 1D ECG signal into 2D patterns of frequency densities, allowing the state-of-the-art techniques of CNNs-based machine learning method to classify AF automatically. It analyzes the time-frequency features of both atrial and ventricular activities, allowing AF detection by using a very short period (five beats) of ECG. By testing it on the AFDB, a performance with 99.41, 98.91, 99.39, and 99.23% for the sensitivity, specificity, PPV, and overall accuracy respectively was achieved, which is superior to the most of the existing algorithms suggesting a great potential in clinical diagnosis in the future.

## Author Contributions

HZ conceived the study. RH performed the design and implementation of the algorithm. QL helped the algorithm design and implementation. RH, HZ, and QL wrote the manuscript. KW and YY commented on and approved the manuscript. NZ and YL contributed to the classification of AF. All authors read and approved the final manuscript.

## Conflict of Interest Statement

The authors declare that the research was conducted in the absence of any commercial or financial relationships that could be construed as a potential conflict of interest.

## References

[B1] Addison P. S. (2005). Wavelet transforms and the ECG: a review. *Physiol. Meas.* 26 R155–R199. 10.1088/0967-3334/26/5/R01 16088052

[B2] Arel I. Rose D. C. Karnowski T. P. (2010). Deep machine learning - a new frontier in artificial intelligence research. *IEEE Comput. Intell. Magazine* 5 13–18. 10.1109/TMI.2018.2833635 29870359

[B3] Artis S. G. Mark R. G. Moody G. B. (1991). Detection of atrial fibrillation using artificial neural networks. *Comput. Cardiol.* 18 173–176. 10.1109/CIC.1991.169073

[B4] Asgari S. Mehrnia A. Moussavi M. (2015). Automatic detection of atrial fibrillation using stationary wavelet transform and support vector machine. *Comput Biol. Med.* 60 132–142. 10.1016/j.compbiomed.2015.03.005 25817534

[B5] Babaeizadeh S. Gregg R. E. Helfenbein E. D. Lindauer J. M. Zhou S. H. (2009). Improvements in atrial fibrillation detection for real-time monitoring. *J. Electrocardiol.* 42 522–526. 10.1016/j.jelectrocard.2009.06.006 19608194

[B6] Censi F. Cianfrocca C. Purificato I. (2013). Atrial fibrillation and the 4P medicine. *Ann. Ist. Super. Sanita* 49 247–248. 10.4415/ANN_13_03_02 24071602

[B7] Cerutti S. Mainardi L. T. Porta A. Bianchi A. M. (1997). Analysis of the dynamics of RR interval series for the detection of atrial fibrillation episodes. *Comput. Cardiol.* 24 77–80. 10.1109/CIC.1997.647834

[B8] Chazal P. D. Reilly R. B. (2006). A patient-adapting heartbeat classifier using ECG morphology and 317 heartbeat interval features. *IEEE Trans. Biomed. Eng.* 53 2535–2543. 10.1109/TBME.2006.883802 17153211

[B9] Couceiro R. Carvalho P. Henriques J. Antunes M. (2008). Detection of atrial fibrillation using model-based ECG analysis. *Int. Conf. Pattern Recog.* 50 1–5. 10.1109/ICPR.2008.4761755

[B10] Dash S. Chon K. H. Lu S. Raeder E. A. (2009). Automatic real time detection of atrial fibrillation. *Ann. Biomed. Eng.* 37 1701–1709. 10.1007/s10439-009-9740-z 19533358

[B11] Daubechies I. (1988). Orthonormal bases of compactly supported wavelets. *Commun. Pure Appl. Math.* 41 909–996. 10.1002/cpa.3160410705

[B12] de Vos C. B. Pisters R. Nieuwlaat R. Prins M. H. Tieleman R. G. Coelen R. J. (2010). Progression from paroxysmal to persistent atrial fibrillation clinical correlates and prognosis. *J. Am. Coll. Cardiol.* 55 725–731. 10.1016/j.jacc.2009.11.040 20170808

[B13] Dokur Z. Olmez T. (2001). ECG beat classification by a novel hybrid neural network. *Comput. Methods Programs Biomed.* 66 167–181. 10.1016/S0169-2607(00)00133-4 11551391

[B14] Gami A. S. Pressman G. Caples S. M. Kanagala R. Gard J. J. Davison D. E. (2004). Association of atrial fibrillation and obstructive sleep apnea. *Circulation* 13 62–63. 10.1161/01.CIR.0000136587.68725.8E 15249509

[B15] García M. Rodenas J. Alcaraz R. Rieta J. J. (2016). Application of the relative wavelet energy to heart rate independent detection of atrial fibrillation. *Comput. Methods Programs Biomed.* 131 157–168. 10.1016/j.cmpb.2016.04.009 27265056

[B16] Goldberger A. L. Amaral L. A. N. Glass L. Hausdorff J. M. Ivanov P. C. Mark R. G. (2000). Physiobank, physiotoolkit, and physionet components of a new research resource for complex physiologic signals. *Circulation* 101 e215–e220. 10.1161/01.CIR.101.23.e215 10851218

[B17] Gomes P. R. Soares F. O. Correia J. H. Lima C. S. (2010). “ECG data-acquisition and classification system by using wavelet-domain hidden Markov models,” in *Proceedings of the Engineering in Medicine and Biology Society (EMBC), 2010 Annual International Conference* Vol. 2010 (Buenos Aires: IEEE), 4670–4673. 10.1109/IEMBS.2010.5626456 21096243

[B18] Guler I. Ubeyli E. D. (2005). ECG beat classifier designed by combined neural network model. *Pattern Recog.* 38 199–208. 10.1016/j.patcog.2004.06.009

[B19] Huang C. Ye S. Chen H. Li D. He F. Tu Y. (2011). A novel method for detection of the transition between atrial fibrillation and sinus rhythm. *IEEE Trans. Biomed. Eng.* 58 1113–1119. 10.1109/TBME.2010.2096506 21134807

[B20] Hubel D. H. Wiesel T. N. (1959). Receptive fields of single neurones in the cat’s striate cortex. *J. Physiol.* 148 574–591. 10.1113/jphysiol.1959.sp006308 14403679 PMC1363130

[B21] Hylek E. M. Go A. S. Chang Y. Jensvold N. G. Henault L. E. Selby J. V. (2003). Effect of intensity of oral anticoagulation on stroke severity and mortality in atrial fibrillation. *N. Engl. J. Med.* 349 1019–1026. 10.1056/NEJMoa022913 12968085

[B22] January C. T. Wann L. S. Alpert J. S. Calkins H. Cigarroa J. E. Cleveland J. C. (2014). 2014 AHA/ACC/HRS guideline for the management of patients with atrial fibrillation: executive summary a report of the American college of cardiology/american heart association task force on practice guidelines and the heart rhythm society. *J. Am. Coll. Cardiol.* 64 2246–2280. 10.1016/j.jacc.2014.03.021 24685669

[B23] Kikillus N. Hammer G. Lentz N. Stockwald F. (2007). Three different algorithms for identifying patients suffering from atrial fibrillation during atrial fibrillation free phases of the ECG. *Comput. Cardiol.* 34 801–804. 10.1109/CIC.2007.4745607

[B24] Kozak C. A. Lawrence J. B. Ruddle F. H. (2008). “Performance analysis of stationary and discrete wavelet transform for action potential detection from sympathetic nerve recordings in humans,” in *in Proceedings of the International Conference of the IEEE Engineering in Medicine and Biology Society 2008*, (Vancouver, BC: IEEE), 2932–2935. 10.1109/IEMBS.2008.4649817 19163320

[B25] Krizhevsky A. Sutskever I. Hinton G. E. (2012). Imagenet classification with deep convolutional neural networks. *Int. Conf. Neural Inf. Process. Syst.* 60 1097–1105.

[B26] Kutlu Y. Kuntalp D. (2012). Feature extraction for ECG heartbeats using higher order statistics of WPD coefficients. *Comput. Methods Programs Biomed.* 105 257–267. 10.1016/j.cmpb.2011.10.002 22055998

[B27] Ladavich S. Ghoraani B. (2015). Rate-independent detection of atrial fibrillation by statistical modeling of atrial activity. *Biomed. Sig. Process. Control* 18 274–281. 10.1016/j.bspc.2015.01.007

[B28] Lake D. E. Moorman J. R. (2011). Accurate estimation of entropy in very short physiological time series: the problem of atrial fibrillation detection in implanted ventricular devices. *Am. J. Physiol. Heart Circ. Physiol.* 300 H319–H325. 10.1152/ajpheart.00561.2010 21037227

[B29] Larburu N. Lopetegi T. Romero I. (2011). Comparative study of algorithms for atrial fibrillation detection. *Comput. Cardiol.* 38 265–268.

[B30] Lecun Y. Bengio Y. Hinton G. (2015). Deep learning. *Nature* 521 436–444. 10.1038/nature14539 26017442

[B31] Lee J. Reyes B. A. Mcmanus D. D. Maitas O. Chon K. H. (2014). Atrial fibrillation detection using an iphone 4s. *IEEE Trans. Biomed. Eng.* 60 203–206. 10.1109/TBME.2012.2208112 22868524

[B32] Lian J. Wang L. Muessig D. (2011). A simple method to detect atrial fibrillation using RR intervals. *Am. J. Cardiol.* 107 1494–1497. 10.1016/j.amjcard.2011.01.028 21420064

[B33] Lin C. H. Du Y. C. Chen T. (2008). Adaptive wavelet network for multiple cardiac arrhythmias recognition. *Exp. Syst. Appl.* 34 2601–2611. 10.1016/j.eswa.2007.05.008 17281539

[B34] Logan B. Healey J. (2005). Robust detection of atrial fibrillation for a long term telemonitoring system. *Comput. Cardiol.* 32 619–622. 10.1109/CIC.2005.1588177

[B35] Mallat S. Zhong S. (1992). Characterization of signals from multiscale edges. *IEEE Trans. Pattern Anal. Machine Intell.* 14 710–732. 10.1109/34.142909

[B36] Mathew S. T. Patel J. Joseph S. (2009). Atrial fibrillation: mechanistic insights and treatment options. *Eur. J. Int. Med.* 20 672–681. 10.1016/j.ejim.2009.07.011 19818285

[B37] Miyasaka Y. Barnes M. E. Gersh B. J. Cha S. S. Bailey K. R. Abhayaratna W. P. (2006). Secular trends in incidence of atrial fibrillation in olmsted county, minnesota, 1980 to 2000, and implications on the projections for future prevalence. *Circulation* 114 119–125. 10.1161/CIRCULATIONAHA.105.595140 16818816

[B38] Moody G. B. Mark R. R. (1983). New method for detecting atrial fibrillation using R-R intervals. *Comput. Cardiol.* 10 227–230.

[B39] Mukamal K. J. Tolstrup J. S. Friberg J. Jensen G. Grønbæk M. (2005). Alcohol consumption and risk of atrial fibrillation in men and women the copenhagen city heart study. *Circulation* 112 1736–1742. 10.1161/CIRCULATIONAHA.105.547844 16157768

[B40] Park J. Lee S. Jeon M. (2009). Atrial fibrillation detection by heart rate variability in poincare plot. *Biomed. Eng. Online* 8 1–12. 10.1186/1475-925X-8-38 20003345 PMC2803479

[B41] Parvaresh S. Ayatollahi A. (2011). “Automatic atrial fibrillation detection using autoregressive modeling,” in *Proceedings of the International Conference on Biomedical Engineering and Technology (ICBET)*, Singapore, 4–5.

[B42] Petrutiu S. Ng J. Nijm G. M. Alangari H. Swiryn S. Sahakian A. V. (2006). Atrial fibrillation and waveform characterization. A time domain perspective in the surface ECG. *IEEE Eng. Med. Biol. Mag.* 25 24–30. 10.1109/EMB-M.2006.250505 17220132

[B43] Potter B. J. Le L. J. (2015). Taking the pulse of atrial fibrillation. *Lancet* 386 113–115. 10.1016/S0140-6736(14)61991-7 25960109

[B44] Rajpurkar P. Hannun A. Y. Haghpanahi M. Bourn C. Ng A. Y. (2017). Cardiologist-level arrhythmia detection with convolutional neural networks. arXiv:1707.01836.

[B45] Rumelhart D. E. Hinton G. E. Williams R. J. (1988). Learning representations by back-propagating errors. *Read. Cogn. Sci.* 323 399–421. 10.1016/B978-1-4832-1446-7.50035-2

[B46] Shyu L. Y. Wu Y. H. Hu W. (2004). Using wavelet transform and fuzzy neural network for VPC detection from the holter ECG. *IEEE Trans. Biomed. Eng.* 51 1269–1273. 10.1109/TBME.2004.824131 15248543

[B47] Slocum J. Sahakian A. Swiryn S. (1992). Diagnosis of atrial fibrillation from surface electrocardiograms based on computer-detected atrial activity. *J. Electrocardiol.* 25 1–8. 10.1016/0022-0736(92)90123-H 1735788

[B48] Tateno K. Glass L. (2001). Automatic detection of atrial fibrillation using the coefficient of variation and density histograms of RR and ΔRR intervals. *Med. Biol. Eng. Comput.* 39 664–671. 10.1007/BF02345439 11804173

[B49] Vollmer M. Sodmann P. Caanitz L. Nath N. Kaderali L. (2017). Can supervised learning be used to classify cardiac rhythms? *Comput. Cardiol.* 44 1–4. 10.22489/CinC.2017.347-176

[B50] Xia Y. Wulan N. Wang K. Zhang H. (2018). Detecting atrial fibrillation by deep convolutional neural networks. *Comput. Biol. Med.* 93 84–92. 10.1016/j.compbiomed.2017.12.007 29291535

[B51] Yaghouby F. Ayatollahi A. Bahramali R. Yaghouby M. (2012). Robust genetic programming-based detection of atrial fibrillation using RR intervals. *Exp. Syst.* 29 183–199.

[B52] Yaghouby F. Ayatollahi A. Bahramali R. Yaghouby M. Alavi A. H. (2010). Towards automatic detection of atrial fibrillation: a hybrid computational approach. *Comput. Biol. Med.* 40 919–930. 10.1016/j.compbiomed.2010.10.004 21051039

[B53] Zhou X. Ding H. Ung B. Pickwellmacpherson E. Zhang Y. (2014). Automatic online detection of atrial fibrillation based on symbolic dynamics and shannon entropy. *Biomed. Eng. Online* 13 1–18. 10.1186/1475-925X-13-18 24533474 PMC3996093

[B54] Zoniberisso M. Lercari F. Carazza T. Domenicucci S. (2014). Epidemiology of atrial fibrillation: european perspective. *Clin. Epidemiol.* 6 213–220. 10.2147/CLEP.S47385 24966695 PMC4064952

